# Hollow Fiber–Supported ZIF-8@GO Reinforced Sol–Gel for Preconcentration of Paraquat Before Determination by UV-Vis Spectrophotometry

**DOI:** 10.1155/jamc/6883692

**Published:** 2025-06-21

**Authors:** Narges Vaezi, Naser Dalali

**Affiliations:** Phase Separation & FIA Lab., Department of Chemistry, Faculty of Science, University of Zanjan, Zanjan, Iran

**Keywords:** HF-SPME, paraquat, sol–gel technology, ZIF-8@GO

## Abstract

A novel, sensitive, and efficient method was developed for the determination of paraquat in soil samples, centered on solid-phase microextraction using hollow fiber–supported ZIF-8@GO reinforced sol–gel combined with UV-Vis spectrophotometry at 257 nm. Silica-based ZIF-8@GO was synthesized through sol–gel technology by reacting tetraethyl orthosilicate (TEOS) with hydrochloric acid (HCl) as a catalyst. The resulting solution was injected into hollow polypropylene fiber segments for in situ gelation. Key microextraction parameters, such as the donor phase pH and volume, stirring rate, extraction time, and desorption conditions (solvent type and volume), were systematically studied and optimized. Under optimal conditions, the method demonstrated linearity within the range of 0.5–2000 μg L^−1^, with a correlation coefficient of 0.999. The relative standard deviation for seven replicate measurements at a concentration of 600 μg L^−1^ paraquat was 0.4%. A preconcentration factor of 631 and a detection limit of 0.15 μg L^−1^ were achieved. Validation of the method was conducted by analyzing soil samples from various sites, yielding recoveries between 96% and 105% with low relative standard deviations, indicating its high accuracy and reliability in detecting trace levels of paraquat.

## 1. Introduction

Paraquat (1,1-dimethyl-4,4-bipyridinium chloride, or PQ) is a commonly utilized chemical herbicide, well-known for its fast-acting and nonselective characteristics. It is additionally categorized as a substance with moderate toxicity [1, 2]. The ionic characteristics and water solubility of this herbicide enable it to easily attach to soil particles, resulting in its immobilization. Soil, an invaluable resource, serves a range of essential functions, from facilitating human activities to maintaining ecosystem balance. In modern mechanized agriculture, the application of pesticides is a widely adopted practice due to its cost-effectiveness, often replacing traditional approaches like manual weed control. While herbicides play a pivotal role in boosting agricultural productivity, their use introduces chemical pollutants into the soil ecosystem, largely originating from production processes. These interactions with soil microorganisms can detrimentally impact soil quality and fertility by altering its physicochemical properties. The degree to which herbicides affect soil microorganisms and their roles depends on various factors, such as the chemical and physical characteristics of the herbicides, soil type, pH, and surrounding environmental conditions. [3]. Various tissues, including the lungs, typically accumulate reactive oxygen species (ROS) and harmful free radicals, playing a significant role in PQ toxicity [4]. Recent research has indicated a link between PQ and Parkinson's disease [5, 6]. Various methods have been used to analyze PQ in natural samples including gas chromatography (GC) [7], gas chromatography-mass spectrometry (GC-MS) [8], high-performance liquid chromatography (HPLC) [9–11], liquid chromatography-mass spectrometry (LC-MS) [12], capillary electrophoresis (CE) [13], and derivatization spectroscopy [14]. Certain sampling methods can be extensive or time-consuming. However, UV-Vis spectrophotometry stands out for its simplicity, speed, versatility, accuracy, and cost-effectiveness [15]. Matrix interferences and low concentrations of PQ often pose challenges in analyzing real samples. Achieving highly sensitive, selective, and precise detection of PQ necessitates the development of effective strategies. As a result, numerous studies have focused on enhancing separation and preconcentration techniques, such as precipitation and coprecipitation, with advancements reported globally [16], cloud point extraction (CPE) [17], liquid–liquid extraction (LLE) [18], and solid-phase extraction (SPE) [19] methods. When selecting methods for separation and preconcentration, significant attention should be directed toward minimizing the use of hazardous solvents by replacing them with environmentally benign alternatives and reducing the complexity of sample preparation procedures. Among the various preconcentration techniques, LLE remains one of the most widely utilized approaches due to its operational simplicity and cost-effectiveness [20]. The process necessitates substantial quantities of organic solvents and is associated with limitations such as repetitive, labor-intensive operations over extended periods, which result in the generation of considerable amounts of hazardous waste [21]. Efforts to address these shortcomings have led to advancements in microextraction techniques, reducing background interference, minimizing sample loss, and enhancing detection limits. [22]. Solid-phase microextraction (SPME) is among the most widely used techniques for microextraction, valued for its speed, simplicity, cost efficiency, and elimination of harmful organic solvents. However, conventional SPME fibers have notable drawbacks, such as fiber breakage, coating degradation, and memory effects. A more recent advancement in this field is hollow fiber SPME, which has garnered considerable attention. This innovative method stands out due to its minimal reliance on organic solvents, ease of operation, and remarkable enrichment efficiency. Unlike traditional SPME, which typically incorporates quartz fibers or stainless steel as supporting materials, this approach uses commercially available polypropylene hollow fibers to contain the adsorbents. The use of hollow fibers offers significant advantages due to their unique properties, including an abundance of micropores extending from the surface to the interior, exceptional mechanical strength, and robust resistance to corrosion. Most importantly, these hollow fibers enhance sample purification by leveraging their microporous walls to effectively block larger molecules and particles from passing through [23]. This method can be applied to a broad spectrum of complex matrix samples. Additionally, the hollow fiber is intended for single-use, effectively preventing cross-contamination and carryover problems [24, 25]. The HF-SPME extraction process operates based on the interaction between the target analytes and the surface functional groups of the sorbent material. Choosing an appropriate sorbent is crucial for significantly enhancing extraction efficiency and effectively isolating target analytes from the sample solution. To date, graphene and graphene oxide (GO) have been widely utilized either as independent sorbents or as supporting platforms for integrating other nanosorbents in hollow fiber–supported SPME, thanks to their remarkable properties [23]. The carbon structure of GO is hexagonal, featuring functional groups like hydroxyl and epoxide on its basal plane, while carboxyl and carbonyl groups predominantly adorn its edges [26, 27]. Their high specific surface area and surface functional groups contributed to the enhanced adsorption capability of the GO sheets. The large delocalized *π* electron system of graphene enables it to generate strong *π*-*π* stacking interactions with benzene rings. GO has been used as an adsorbent in SPME in recent years [28], magnetic solid-phase extraction (MSPE) [29], dispersive solid-phase extraction (DSPE) [30], and GO-reinforced HF [31]. Incorporating GO into the extractor phase improves the sorbent's capacity for extraction. In order to create GO-reinforced HF, researchers have been concentrating on metal–organic frameworks (MOFs), one of the artificial sorbents coated on GO. Based on the coordination of metal ions or clusters, crystallized porous coordination polymers, or MOFs, have stiff and symmetric organic linkers that form very porous crystalline networks [32]. A type of MOF called zeolitic imidazolate frameworks (ZIFs) offers increased flexibility and hydrostatic stability. The coordination of N atoms within the imidazolate anion and metallic ions, like Zn^2+^ or Co^2+^ [33, 34], is the basis of ZIFs. Recently, ZIF-8, which belongs to a subclass of ZIFs, has been selected as a promising candidate. ZIF-8 has a chemical formula called Zn (Hmim)_2_, which contains 2 MIM and the metal atom Zn. The robust bonding structure of ZIF-8 is responsible for its superior chemical and hydrothermal stability. The thermal stability of ZIF-8 is demonstrated by its structural stability being maintained at temperatures equal to 500°C. Furthermore, ZIF-8's porosity and crystallinity are preserved even after it has been dissolved in water or organic solvents [35]. The incorporation of ZIF-8 particles into GO sheets can enhance control of ZIF-8 particles (structure, morphology, etc.) and prevent their accumulation and leaching out of the membrane lattice. The coordination of metal particles and COOH groups results in the equilibration of MOF particles on GO surface [36, 37]. Our study demonstrates the development of an innovative, straightforward, sensitive, and highly specific SPME technique that uses a hollow fiber–supported sol–gel system combined with ZIF-8@GO. This approach was able to detect PQ in soil samples with a significant preconcentration factor through UV-Vis spectrophotometry. The research was focused on improving existing methods by introducing a simple and practical solution that tackles the persistent challenges of conventional SPME fibers, such as sample carryover. The reaction of tetraethyl orthosilicate (TEOS) with an acidic catalyst (HCl) was utilized in the manufacture of a silica-based organic–inorganic polymer containing ZIF-8@GO using sol–gel technology. After injecting this solution into a polypropylene hollow fiber segment, the gel formation process was carried out on site. The disposable device was utilized in direct immersion sampling mode during sample analysis. The sorbent's stability is enhanced and its loss from the hollow fiber surface is minimized by the chemical bonding formed during the sol–gel procedure. Additionally, dispersing the sorbent into the sol solution facilitates the creation of smaller sorbent particles within the hollow fiber pores, which enhances the sorbent's surface area and boosts analyte extraction efficiency. By generating open pathways within the hollow fiber structure, the sol–gel technique improves mass transfer of the analyte into the fiber network, leading to a substantial reduction in extraction time [38]. The affecting parameters on the extraction of the analyte such as pH, extraction time, stirring rate, and sample volume were systematically investigated and optimized by a univariable method, and the best conditions were chosen. In the end, the proposed method was validated through evaluations of linearity, detection limits, precision, reproducibility, and accuracy. The application of it was successful in extracting and quantifying PQ from various matrices.

## 2. Experimental

### 2.1. Reagents and Materials

The chemicals used in this work were all of the highest purity and were not further purified. Double distilled water was used for all dilutions. The chemicals used were graphite powder mesh 320 (99% w/w), H_2_SO_4_ (98% w/w), KMnO_4_ (99% w/w), H_2_O_2_ (35% w/w), 2-methylimidazole (2-MIm, 99% w/w), zinc nitrate hexahydrate (Zn (NO_3_)_2_·6H_2_O, 99% w/w), and methanol (CH_3_OH, 99.9% w/w) for the synthesis of ZIF-8@GO, Sodium chloride (NaCl, 99.5% w/w), and Potassium chloride (KCl, 99% w/w) for the investigation of the salt effect, Sodium hydroxide (NaOH, 0.1 mol L^–1^) and hydrochloride acid (HCl, 0.1 mol L^–1^) to adjust the pH of the solutions in the whole study, all of which were procured from (Merck, Darmstadt, Germany). The stock solutions (1000 μg mL^−1^) of PQ were prepared by direct dissolution of proper amounts of PQ dichloride ((C_6_H_7_N)_2_) Cl_2_, 95% w/w, Aria Shimi, Iran) with double distilled water in a 100 mL flask. The working standard solutions were prepared by diluting stock standard solutions. The stock Q3/2 polypropylene hollow fiber membrane (200 μm wall thickness, 600 μm inner diameter, and 0.2 μm average pore size) was obtained from Membrana GmbH (Wuppertal, Germany).

### 2.2. Instrumentation

All the absorbance measurements were performed with a double beam UV-Vis spectrophotometer model Specord 210 plus (Germany) through a semi-micro rectangular cell (Starna, USA) with a path length of 10 mm. An infrared spectrometer (Thermo, UK) was used to record FTIR spectra of the as-prepared ZIF-8 and its composite for the identification of functional groups within the range of 400–4000 cm^−1^. A Bruker-DB X-ray diffractometer (Germany) with Cu Kα radiation (*λ* = 1.540 Å) was employed for recording the X-ray diffraction pattern (XRD) of products. The surface morphology of the synthesized adsorbent was studied via field emission scanning electron microscopy (FESEM) using the model ( Mira3-XMUTescon, Czech Republic).

### 2.3. Preparation of Real Sample

Samples of soil were taken from farms in Sain Qaleh city (Zanjan Province, Iran). 10 g of soil, 30 mL of acetone, and 20 mL of water were combined to prepare soil samples, which were then stirred for 30 min at 600 rpm using a magnetic stirrer and filtered through Whatman filter paper (WHA 1442042).

### 2.4. Synthesis of GO

GO was prepared using a modified Hummers method. The GO synthesis procedure is described as follows: graphite (1 g) and concentrated sulfuric acid (H_2_SO_4_, 23 mL) were added into a 500 mL flask in an ice bath under continuous stirring for 60 min. Potassium permanganate (KMnO_4_, 3 g) was slowly added to the suspension after a while, and the reaction mixture was kept at 0°C while stirring continuously. After 15 min, the flask was removed from the ice bath and placed inside the oil bath for 120 min at a temperature of 40°C. In the next step, 46 mL of deionized water was added to the mixture, and stirring continued for 120 min at 40°C. The reaction vessel was removed from the oil bath and 100 mL of deionized water and 10 mL of H_2_O_2_ were added to the mixture above. In this case, the color of the solution changed from dark brown to light brown. The mixture was filtered and washed with a 1:10 HCl aqueous solution (250 mL) for several cycles and separated by centrifugation until a neutral pH was achieved. The product was dried in an oven at 60°C for 2 h [39].

### 2.5. Synthesis of ZIF-8@GO

The ZIF-8@GO composite was prepared by modifying the ZIF-8 synthesis procedure described in the literature. Briefly, Zn (NO_3_)_2_ (0.366 g) was dissolved in 12 mL of methanol. Separately, ligand solution was prepared by dissolving 2-MeIM (0.811 g) in methanol (20 mL). 0.366 g of GO was dispersed into the ligand solution and sonicated for 30 min. A greyish mixture quickly formed after the Zn solution was gradually added to the ligand solution while being stirred continuously for 3 hours. The precipitate was collected by centrifugation, and it was then three times washed with methanol and dried in an oven at 80°C for at least 7 hours [40, 41].

### 2.6. Preparation of ZIF-8@GO/SiO_2_ Reinforced Hollow Fiber

An acid-based catalytic method [42, 43] was used to prepare the sol solution of the functionalized ZIF-8@GO/silica composite. One of the advantages of the sol–gel method is the possibility of using different precursors. In this work, TEOS was used as the precursor. The process of sol–gel involves two primary reactions, one of which is the precursor's hydrolysis and the other is the polycondensation of the hydrolysis product. Sols were created by adding TEOS, HCl solution, ethanol (EtOH), and Triton X-100 to a solution through an acid-based catalyzed approach. The glass vial (15.2 cm^3^) was filled with TEOS and EtOH in equal proportions and agitated for 10 min. Subsequently, a 1 mL concentrated HCl solution was added, and the mixture was shaken to promote the condensation and hydrolysis processes. Following a 15 min duration, the sol was mixed with 300 μL of Triton X-100 and left to stir for a further 120 min. A ZIF-8@GO composite sol solution was created by mixing 1 mL of silica sol with 5 mg of ZIF-8@GO after 30 min of stirring. The hollow fibers were cut into 2 cm pieces by hand and cleaned with acetone to remove impurities before drying. The sol solution was finally injected into the hollow fiber's lumen with a microsyringe at a slow pace. The fibers were left to dry during ambient conditions. Both ends of the fibers were sealed to prevent leakage. After preparation, the fibers were inserted into a glass vial (15.2 cm^3^), where they were soaked with 10 mL of an analyte solution with an optimum pH level. The content was stirred at 700 rpm magnetically for 30 min. At the right extraction time, the porous hollow fiber permits the analyte to diffuse from the solution into the sorbent. After removing the hollow fiber, it was placed in 0.5 mL of methanol as a desorption solvent in a closed vial, which allowed the analyte to absorb from the adsorbent. UV-Vis spectrophotometry was used to measure the extracted analyte.

## 3. Results and Discussion

### 3.1. Characterization of GO and ZIF-8@GO as Synthesized Adsorbents

**Figure 1:**
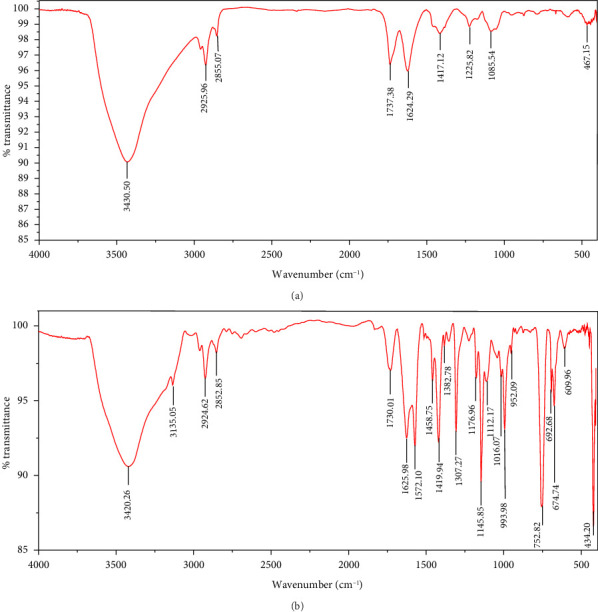
FTIR spectra of (a) GO and (b) ZIF-8@GO.

**Figure 2:**
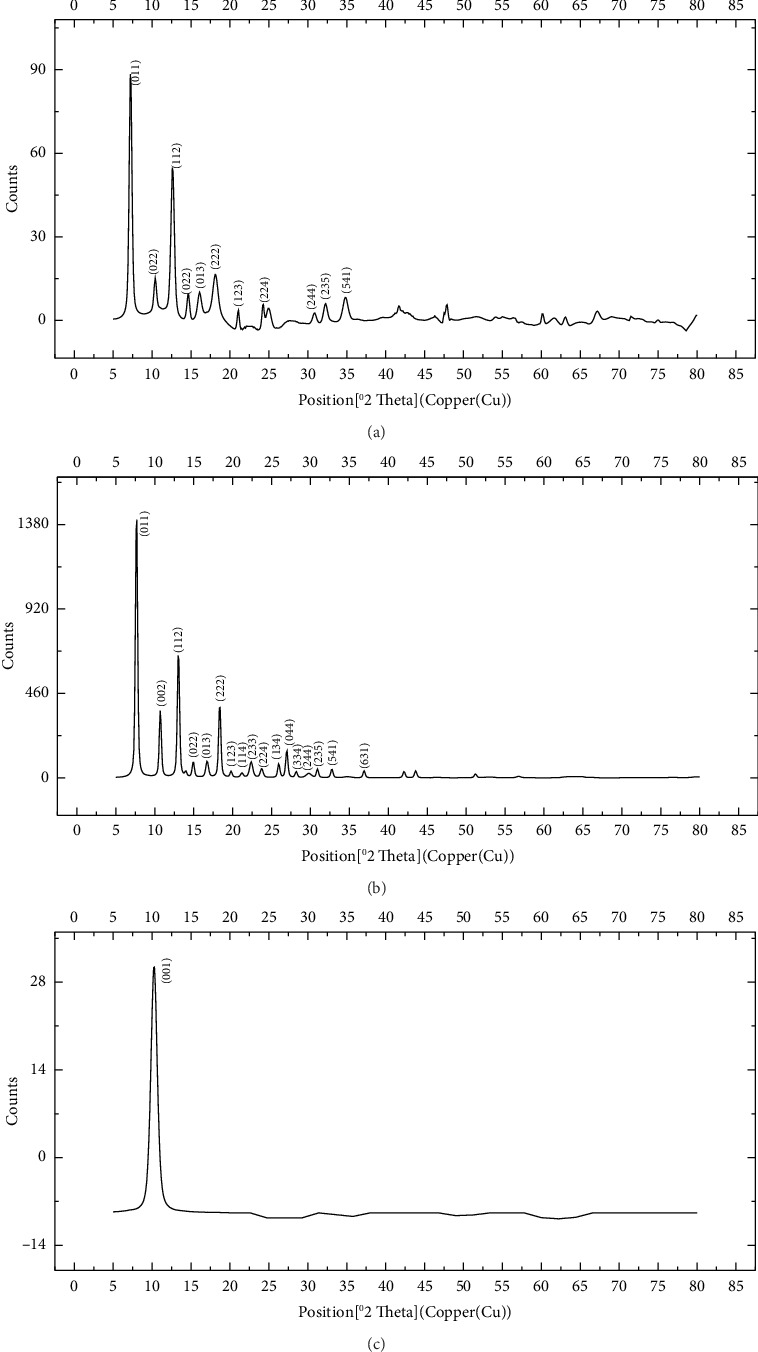
XRD pattern of (a) ZIF-8@GO, (b)ZIF-8, (c) GO.

**Figure 3:**
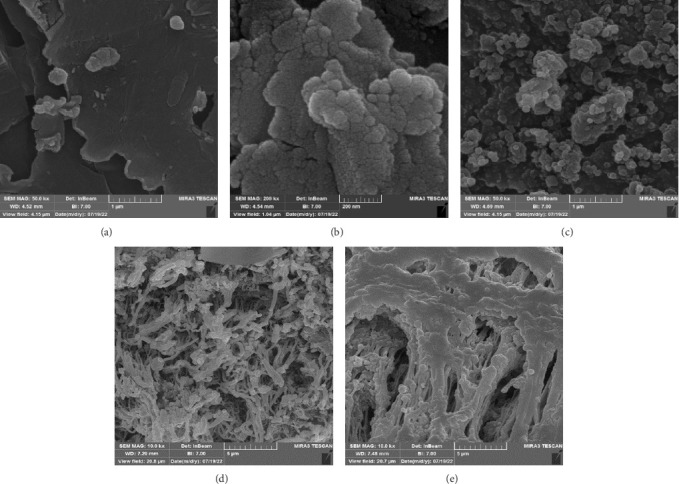
FESEM of (a), (b) GO, (c) ZIF-8@GO, (d) HF, and (e) modified HF.

The FTIR spectroscopy of GO (Figure 1(a)) revealed several functional groups. The presence of OH functional groups in the structure can be confirmed by a highly intense band at 3430 cm^−1^. Very weak bands at 2855 cm^−1^ and 2925 cm^−1^ were due to the symmetric and asymmetric stretching vibrations of the C-H bond, respectively. Carbonyl C=O stretching vibration (1737 cm^−1^), C=C stretching vibration of unoxidized graphitic domain (1624 cm^−1^), O–H deformation (1417 cm^−1^), C–O stretching of epoxy groups (1225 cm^−1^), C–O stretching vibration of alkoxy group (1085 cm^−1^), and the bending vibration outside of plane O-H band (467 cm^−1^) were also observed in the FTIR spectra of GO. The presence of oxygen-containing functional groups like hydroxyl, epoxy, carboxyl, and carbonyl within the GO structure was confirmed by the FTIR result [44]. FTIR spectra of ZIF-8@GO (Figure 1(b)) showed a band at 3420 cm^−1^ due to O-H stretching vibration indicating the presence of OH functional group, and aromatic C-H stretching vibration band of imidazole is observed at 3135 cm^−1^. The weak bands at 2852 cm^−1^ and 2924 cm^−1^ were due to the symmetric and asymmetric stretching vibration of the C–H bond, respectively, like GO. Stretching vibrations of the carbonyl and unoxidized C=C groups can be identified by the bands at 1730 cm^−1^ and 1625 cm^−1^. The existing bands at 1572 cm^−1^ and 1458 cm^−1^ −1016 cm^−1^ are related to the C=N and C-N bonds of imidazole, respectively. The bands at 893 cm^−1^, 752–674-674 cm^−1^, and 434 cm^−1^ are related to the imidazole ring (C=C, vibrations) and Zn-N, respectively. The Zn-N stretching vibration observed at 434 cm^−1^ shows that Zn^2+^ ions have been successfully coordinated with imidazole, which is in line with the characteristic framework structure of ZIF-8 [45]. The GO XRD pattern is shown in Figure 2(a). Graphite's oxidation is the main reason for the diffraction peak at 2*θ* = 10.2°. The absence of any other peaks except for 10.2° indicates that there were no impurities in the final product. The pattern of ZIF-8@GO (Figure 2(c)) is similar to pristine ZIF-8 (Figure 2(b)) with the difference that the intensity of the peaks has been reduced, which can be presumed that the secondary species (GO) interfere and reduce the intensity of the peaks. Field emission scanning electron microscopy was used for the initial analysis of the surface morphology of GO. Figures 3(a) and 3(b) reveal a single layer of GO. The surface of GO sheets is relatively large and can be easily identified. The structure of GO is made up of layers with a relatively smooth surface, wavy edges, and slight wrinkles. Sheets of GO create a porous network that looks like a loose sponge with an approximate size of 45.43 nm. The FESEM image of the ZIF-8@GO composite (Figure 3(c)) shows that ZIF-8 nanoparticles are distributed on the surface of GO nanosheets, indicating that ZIF-8 is successfully grown on the surface of GO. In Figures 3(d) and 3(e), FESEM images of HF and modified HF are displayed. It is evident that ZIF-8@GO has successfully been attached to the hollow fiber's lumen by comparing the two figures.

### 3.2. Determination of the pH of the Point of Zero Charges (pHpzc)

Aliquots of 10 mL of NaNO_3_ solutions at a concentration of 0.1 mol L^−1^ and various initial pH values (2 < pH < 10) were stirred with 10 mg of ZIF-8 for 24 h at room temperature in order to determine the pHpzc, which denotes the pH value at which the electric charge on the ZIF-8@GO surface is zero. Solutions of NaOH and HCl at 0.1 mol L^−1^ were used to balance the pH. Before and after the agitation process, the pH values of the solution were measured, and a plot of ΔpH (pHf-pHi) versus initial pH was created. The obtained results indicate that ZIF-8@GO's zero charge point is equivalent to 7. The pHpzc value of seven is consistent with previously reported values for ZIF-8-based composites, which typically range from 7 to 9.8, indicating that the functionalization with GO did not significantly alter the surface charge characteristics of the material.

### 3.3. Effect of the Amount of ZIF-8@GO

**Figure 4:**
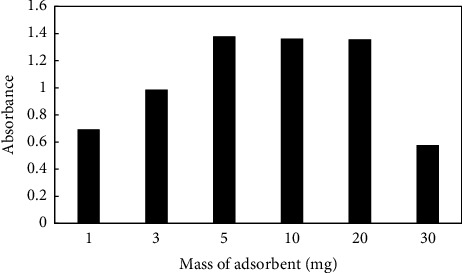
Effect of the amount of ZIF-8@GO. 10 mL of a sample containing 2 mgL^−1^ paraquat at pH 7, stirring rate: 500 rpm, time of extraction: 30 min, and mass of adsorbent: variable. Elution condition: 0.5 mL methanol; elution time: 30 min.

Amounts of 1, 3, 5, 10, 20, and 30 mg of ZIF-8@GO were added to the sol–gel solutions in order to determine the effect of the adsorbent quantity on the extraction efficiency. The findings in Figure 4 demonstrated that selecting 5 mg of ZIF-8@GO had a beneficial effect on the amount of PQ that was extracted, and that effect was maintained up to 20 mg. When more than 20 mg was used, the absorbance signal is decreased when using more than 20 mg. The decrease is caused by adsorbent particles obstructing the fiber pores, and surface interactions between the analyte and the adsorbent species are thereby reduced.

### 3.4. Effect of pH

**Figure 5:**
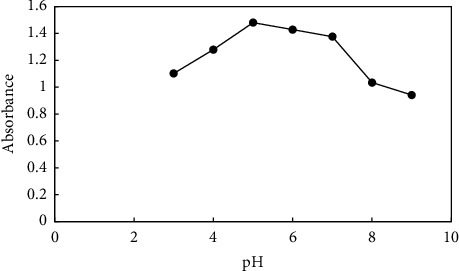
Effect of pH. Extraction conditions: 10 mL of a sample containing 2 mgL^−1^ paraquat at pH variable, stirring rate: 500 rpm, time of extraction: 30 min, and mass of adsorbent: 5 mg. Elution condition: 0.5 mL methanol; elution time: 30 min.

The effect of pH on the extraction of PQ was examined, and the results are illustrated in Figure 5. The pH of the solution was examined by adjusting it with either HCl or sodium hydroxide between 3 and 9. According to the results, at pH 3–5, the absorbance signal increases and then diminishes, so pH 5 was chosen for all experiments. Due to the fact that paraquat is stable in acidic and neutral situations and annihilate in alkaline situations; Considering the point of zero at pH = 7 and the positivity of the adsorbent in aforementioned region and the cationic nature of paraquat, which has the maximum absorbance signal at pH = 5, it cannot be considered electrostatic interaction. Rather, it can be (i) hydrogen bonding between hydrogen atom of paraquat and nitrogen atom of 2-methyl imidazolate ZIF(8) and hydrogen of paraquat and oxygen of hydroxyl and carboxyl groups on the surface of graphene oxide (ii) π-π interaction between paraquat pyridine ring and aromatic ring of graphene oxide (iii) CH-π and cation-π interactions.

### 3.5. Effect of the Stirring Rate

**Figure 6:**
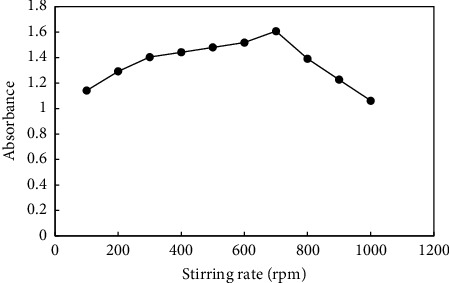
Effect of the stirring rate. Extraction conditions: 10 mL of a sample containing 2 mgL^−1^ paraquat at pH 5, stirring rate: variable, time of extraction: 30 min, and mass of adsorbent: 5 mg. Elution condition: 0.5 mL methanol; elution time: 30 min.

In HF-SPME, using a sufficiently high stirring rate can significantly reduce the extraction time. An increased stirring rate enhances the mass transfer rate of the analyte from the aqueous phase to the membrane while simultaneously decreasing the thickness of the boundary layer at the outer surface of the membrane. This, in turn, it accelerates the extraction kinetics. Diverse stirring rates (100–1000 rpm) were tested to determine their impact on the preconcentration of PQ. It was observed in Figure 6 that the absorbance signal decreased at a stirring rate of over 700 rpm due to mechanical stress on the fiber. Thus, 700 rpm was chosen as the optimal stirring speed.

### 3.6. Effect of the Time of Extraction

**Figure 7:**
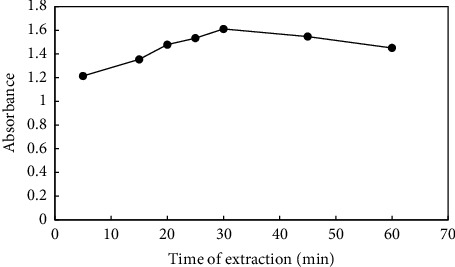
Effect of the time of extraction. Extraction conditions: 10 mL of a sample containing 2 mgL^−1^ paraquat at pH 5, stirring rate: 700 rpm, time of extraction: variable, and mass of adsorbent: 5 mg. Elution condition: 0.5 mL methanol; elution time: 30 min.

The distribution of the target analyte between the sample solution and the adsorbent is influenced by extraction time, which is crucial in the HF-SPME process. Additionally, since HF-SPME may function as an equilibrium-based method, there is a clear correlation between the mass transfer of analytes from the sample solution to the sorbent and the duration of the extraction. To achieve the highest microextraction productivity, the extraction time was examined in this way. The porosity of sol–gel is a significant factor in the equilibration time of analyte extraction. The extraction time depends on the thickness and porosity of the chosen sorbent. Thicker sorbents have a longer diffusion time for the analyte than thinner ones. The diffusion of analyte through a thin porous coating is faster than through a thick, nonporous one. The sol–gel network is porous, which compensates for the impact of sorbent thickness. At room temperature, the extraction efficiency of PQ increased up to 30 min after evaluating different extraction times from 5 to 60 min. After this time, the absorbance signal diminished as a result of the decrease in active sites on the adsorbent interacting with the analyte species. In this manner, an extraction time of 30 min was chosen as the ideal time (Figure 7).

### 3.7. Effect of Ionic Strength

In order to examine the impact of ionic strength on the performance of HF-SPME, varying amounts of NaCl and KCl (0.1–1, mol L^−1^) were used in different experiments. The results show that the absorbance declined as the salt concentration in the aqueous sample increased. Subsequent extractions do not require the addition of salt to the sample solution. The explanation of this issue may be related to the change in the physical properties of the Nernst diffusion layer, which leads to the reduction of the diffusion of analyte from the sample to the receiving phase. Electrostatic interaction between the polar analyte and salt particles can lead to a decrease in the absorbance signal due to the reduction in transfer to the acceptor phase.

### 3.8. Effect of Donor Phase Volume

**Figure 8:**
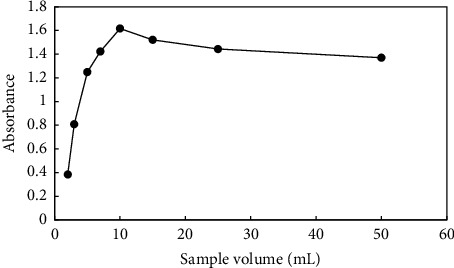
Effect of donor phase volume. Extraction condition: various volumes of a sample containing 2 mgL^−1^ paraquat at pH 5. Stirring rate: 700 rpm; time of extraction: 30 min. Elution condition: 0.5 mL methanol; elution time 30 min.

An effective parameter for extraction efficiency is sample volume. The preconcentration factor in the microextraction process can generally be improved by increasing the volume ratio of the donor phase to the acceptor phase. Different volumes of the donor phase (2–50 mL) were utilized to evaluate this effect while the acceptor phase remained constant (Figure 8). The extraction efficiency and preconcentration factor were increased with the donor phase volume being increased from 2 to 10 mL. Extraction efficiency and absorbance decreased with an increase in sample volume. This phenomenon could be caused by the poor mass transfer kinetics and saturation of the sorption capacity [46]. Thus, a sample volume of 10 mL was chosen for the subsequent studies.

### 3.9. Desorption Studies

Desorption of PQ from the sorbent plays a crucial role in improving the method of measuring the analyte. To attain a high preconcentration factor, the eluting solution must be able to completely desorb the analyte with the least volume and must not interfere with the determination of the analyte. For this purpose, four types of protic and aprotic solvents (methanol, ethanol, acetone, and acetonitrile) with different polarities that could desorb PQ from the fiber without affecting the structure of PQ and without dissolving the propylene membrane were investigated. According to the results, methanol was selected as an eluting solvent. The next step involved optimizing the eluent volume by desorbing the analyte with different methanol volumes (0.5–2.0 mL). It was found that 0.5 mL of methanol was selected for the complete desorption of PQ because subsequent additions of methanol did not result in a measurable increase in the absorbance of the collected eluent.

### 3.10. Method Validation

The analytical figures of merit of the method were examined in order to verify the method's suitability for the analysis of PQ and to validate its performance characteristics. Under the optimal extraction conditions, the following figures of merit are examined: precision, linear dynamic range (LDR), limit of detection (LOD), limit of quantification (LOQ), and coefficient of determination (*R*^2^). The linear ranges and coefficients of determination of the target analyte were satisfactory. The calibration curve was obtained using a series of standard solutions containing the PQ at 19 different concentrations. The HF-SPME procedure that was proposed had good linear behavior within the range of 0.5–2000 μg L^−1^ and an acceptable coefficient of determination of 0.9992. The LOD (based on S/N = 3) and LOQ (based on S/N = 10) were found to be 0.15 and 0.5 μg L^−1^, respectively. The relative standard deviation of the method using six replicate measurements of the standard solution at a concentration level of 600 μg L^−1^ was obtained as 0.48%. The preconcentration factor of 631 was calculated using the following equation:

(1) $$\begin{array}{c} \text{PF}=\frac{A_{f}}{A_{\text{in}}}\times \frac{V_{\text{in}}}{V_{e}}, \end{array}$$

where PF is the preconcentration factor, *A*_*e*_ and *A*_in_ represent the absorbance of the eluent and the initial solution, and *V*_in_ and *V*_*e*_ are the initial sample volume and elution solvent volume, respectively.

### 3.11. Effect of Foreign Compounds

To evaluate the procedure's selectivity and ability, it was carried out in the presence of various pesticides (2 and 5 mgL^−1^). Any deviation of ±5% or more from the absorbance value of the standard solution was considered interference [47, 48]. The results shown in Table 1 indicate that these compounds did not show any significant interference in the determination of paraquat, and since they have no absorbance signal, the method is completely selective.

### 3.12. Real Sample Analysis

The method's performance was tested by analyzing PQ in different soil samples. Following real sample preparation (Section 2.3), to assess the accuracy of the method, three known concentration levels of PQ (1, 10, and 100 μg L^−1^) were spiked to the samples and the relative recoveries were calculated for the three replicate analyses. The results are shown in Table 2.

The relative recovery (RR%) was calculated by the following equations:

(2) $$\begin{array}{c} \text{RR}\%=\left[\frac{\left(C_{\text{found}}-C_{\text{initial}}\right)}{C_{\text{added}}}\right]\times 100. \end{array}$$

Relative recoveries were in the range of 96.0%–105.0% with RSDs between 0.004% and 1.25% (*n* = 3). The results indicated that the extraction effectiveness of the approach was not significantly affected by complicated matrices and the proposed method could be considered as a useful tool for the quantification of PQ in soil samples.

### 3.13. Method Comparison

A comparison between the proposed method and other preconcentration techniques for extracting and quantifying PQ in biological and environmental samples is provided in Table 3. The proposed method exhibits several notable advantages, including an extensive LDR, low LOD and LOQ, and a high preconcentration factor. Additionally, the hollow fiber segment serves as an independent device, facilitating direct application for extraction. Its straightforward usability and absence of memory effects further enhance its practicality. The proposed method is highly suitable for routine laboratory analyses of PQ in soil samples based on these characteristics.

### 3.14. Conclusion

For the preconcentration and identification of PQ in soil samples, a contemporary, straightforward, sensitive, and selective hollow fiber SPME technique in conjunction with UV-Vis spectrophotometry was created. Using this technique, ZIF-8 was synthesized and incorporated into the surface of GO nanosheets. ZIF-8@GO was created using the sol–gel technique and injected into the lumen of the hollow fiber. The aforementioned composite benefited from a high interaction between the sorbent and analyte. Through channels in the polypropylene walls, the analyte molecules (in the feed solution) and the adsorbent (sol–gel inside the fiber) come into contact with one another. Because of the holes, analyte molecules may display some form of dimensional selectivity. This method's benefits over other common SPME fibers include its versatility, ease of use, high preconcentration factors, and easier handling. Hollow fiber that is only used once can lessen the possibility of cross-contamination and carryover issues. The suggested approach satisfies factory relative standard deviations and yielded low LOD and very wide LDR under optimal conditions. One drawback of this process is that automation is not yet simple and convenient to implement.

## Figures and Tables

**Table 1:** Effect of foreign compounds on the determination of PQ (2 mgL^−1^).

Contaminants	Concentration (mgL^−1^)	Absorbance (PQ) ± SD (*n* = 3)^∗^
Diazinon	0	1.6187 ± 0.0380
	2	1.6125 ± 0.0186
	5	1.5831 ± 0.0084

Ethion	0	1.6187 ± 0.0380
	2	1.6031 ± 0.0155
	5	1.5941 ± 0.0121

Fenitrothion	0	1.6187 ± 0.0380
	2	1.6078 ± 0.0223
	5	1.5997 ± 0.0006

Methyl parathion	0	1.6187 ± 0.0380
	2	1.6043 ± 0.0221
	5	1.5524 ± 0.0002

Bentazon	0	1.6187 ± 0.0380
	2	1.6076 ± 0.0254
	5	1.5735 ± 0.0005

^∗^Absorbance signal of paraquat for three replicate analyses ± standard deviation.

**Table 2:** Determination of paraquat in soil samples.

Sample	Added (μg L^−1^)	Found (μg L^−1^)	Recovery (%)	RSD (%)^∗^
Soil 1	0	7	—	—
	1	7.99	99	1.25
	10	16.8	98	0.25
	100	106.8	99.8	0.0181

Soil 2	0	—	—	—
	1	1.05	105	1
	10	9.98	99.8	0.65
	100	96.9	96	0.043

Soil 3	0	10	—	—
	1	10.998	99.8	0.2
	10	19.97	99.7	0.078
	100	109	99	0.004

^∗^RSD: relative standard deviation percent.

**Table 3:** Comparison of the proposed method with some other methods for determination of paraquat.

Matrix	Extraction method	^g^LDR (μg L^−1^)	^h^LOD (μg L^−1^)	^i^LOQ (μg/L)	^j^PF	^k^RSD (%)	Ref.
Environmental/biological samples	^a^IP-SHS-HLLME/HPLC	0.5–500	0.2	0.5	74	< 5	[49]
Human plasma/urine	^b^MDSPE/HPLC	28.5–570.2	4.5	15.2	40	2.3	[50]
Plasma and urine samples	^c^SPE/UV-Vis	15.0–400.0	12.2	—	35	0.65	[51]
Water samples	SPE/^d^CE	2.5–100	1.95	—	166.66	2.86	[52]
Urine sample	MDSPE/^e^HPLC-MS	3.75–375	0.94	2.82	—	< 3.25	[53]
Soil sample	IPSBME	1–2000	0.3	1	355.34	0.6	[54]
Soil samples	^f^HF-SPME/UV-Vis	0.5–2000	0.15	0.5	631	0.4	This work

^a^Ion-pair switchable-hydrophilicity solvent–based homogeneous liquid–liquid microextraction coupled to high-performance liquid chromatography.

^b^Magnetic dispersive solid-phase extraction and high-performance liquid chromatography.

^c^Solid-phase extraction and UV-Vis spectrometry.

^d^Capillary electrophoresis.

^e^High-performance liquid chromatography-tandem mass spectrometry.

^f^Hollow fiber solid-phase microextraction.

^g^Linear dynamic range.

^h^Limit of detection.

^i^Limit of quantification.

^j^Preconcentration factor.

^k^Relative standard deviation.

## Data Availability

The majority of the data used to support the findings of this study are included in the article. Other data are available from the corresponding author upon request.
