# Correction to “Hollow Fiber–Supported ZIF‐8@GO Reinforced Sol–Gel for Preconcentration of Paraquat Before Determination by UV‐Vis Spectrophotometry”

**DOI:** 10.1155/jamc/9874058

**Published:** 2026-01-18

N. Vaezi and N. Dalali, “Hollow Fiber–Supported ZIF‐8@GO Reinforced Sol–Gel for Preconcentration of Paraquat Before Determination by UV‐Vis Spectrophotometry,” *Journal of Analytical Methods in Chemistry* 2025, no. 1 (2025): 1–13, https://doi.org/10.1155/jamc/6883692.

In the article titled “Hollow Fiber–Supported ZIF‐8@GO Reinforced Sol–Gel for Preconcentration of Paraquat Before Determination by UV‐Vis Spectrophotometry,” the details for the corresponding author were incorrect.

Correspondence should now be addressed to Naser Dalali, naser.dalali@znu.ac.ir.

The above change is due to Dr. Dalali’s contributions during article revision and response to reviewers’ questions during peer review. Both authors agree to this change.

We apologize for this error.

